# Integrative analysis of TROAP with molecular features, carcinogenesis, and related immune and pharmacogenomic characteristics in soft tissue sarcoma

**DOI:** 10.1002/mco2.369

**Published:** 2023-09-18

**Authors:** Chao Tu, Binfeng Liu, Chenbei Li, Chengyao Feng, Hua Wang, Haixia Zhang, Shasha He, Zhihong Li

**Affiliations:** ^1^ Department of Orthopaedics The Second Xiangya Hospital of Central South University Changsha Hunan China; ^2^ Hunan Key Laboratory of Tumor Models and Individualized Medicine The Second Xiangya Hospital of Central South University Changsha Hunan China; ^3^ Shenzhen Research Institute of Central South University Guangdong China; ^4^ Department of Oncology The Second Xiangya Hospital of Central South University Changsha Hunan China

**Keywords:** malignant phenotype, prognosis, soft tissue sarcoma, TROAP, tumor immune infiltration

## Abstract

Soft tissue sarcoma (STS) is an uncommon malignancy that often carries a grim prognosis. Trophinin‐associated protein (TROAP) is augmented in a variety of tumors and can affect tumor proliferation. Nevertheless, the prognostic value and specific functions of TROAP in STS are still vague. Herein, we display that TROAP exhibits an augmented trend in STS, and its elevation correlates with a poor prognosis of STS. Furthermore, its reduction is related to increased immune cell infiltration, enhanced stroma, and elevation of immune activation. Meanwhile, the TROAP‐derived genomic signature is validated to predict patient prognosis, immunotherapy, and drug response reliably. A nomogram constructed based on age, metastatic status, and a TROAP‐derived risk score of an STS individual could be used to quantify the survival probability of STS. In addition, in vitro experiments have demonstrated that TROAP is overexpressed in STS, and the downregulation of TROAP could affect the proliferation, migration, metastasis, and cell cycle of STS cells. In summary, the TROAP expression is elevated in STS tissues and cells, which is related to the poor prognosis and malignant biological behaviors of STS. It could act as a potential prognostic biomarker for diagnosis and treatment of STS.

## Graphical abstract

Herein, we comprehensively explored the molecular characteristics and prognostic value of TROAP in soft tissue sarcoma (STS), revealing its relationship with the immune microenvironment and chemotherapy. Also, we confirmed that TROAP is elevated in STS and demonstrated its capacity to regulate the proliferation, migration, invasion, and cell cycle of STS cells. These findings provide a new perspective for assessing prognosis, tailoring individualized treatments, and studying the pathogenic mechanisms of STS (Created with BioRender.com).

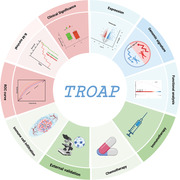

## 1 INTRODUCTION

Soft tissue sarcoma (STS) is a general term for a class of malignant tumors arising from mesenchymal tissues throughout the body.^1^ STS typically present as asymptomatic masses, mostly in the retroperitoneum, internal organs, and extremities. This disease is characterized by low incidence, high recurrence rate, complex pathological types, and significant heterogeneity in biological specificity and histological manifestations.^2^, ^3^ Since the 1970s, the prognosis of STS has been somewhat alleviated when surgery combined with chemotherapy became the standard therapeutic strategy.^3^ However, recurrence and metastasis still occur in 20−30% of the STS cohort, and their 5‐year survival rate remains below 30%.^4^, ^5^ Currently, effective treatment measures for STS patients with advanced, metastatic, and recurrent are still limited, apart from chemotherapy, antiangiogenic drugs, and a few specific targeted drugs. Therefore, early diagnosis, exploration of prognostic biomarkers, and precise individualized treatment are of great significance in improving the clinical outcome of patients with STS.

Tropinin‐associated protein (TROAP), also known as tastin, is a cytoplasmic protein discovered in human epithelial cells by Fukuda et al.^6^ in 1995, which consists of 778 amino acid residues and contains potential protein kinase phosphorylation sites. TROAP was originally described as a soluble cytoplasmic protein and serves as an accessory protein of trophoblasts involved in early embryo implantation by mediating cell invasion and proliferation.^6^ Also, it is abundant in testis, bone marrow and thymus tissue.^7^ Meanwhile, previous studies have proved that TROAP is an essential protein for the function of trophin as a cell adhesion molecule and is relevant to human embryo implantation and endometrial formation.^8^ Moreover, it has been reported that TROAP is associated with the organization of the microtubule cytoskeleton and is required for assembling the bipolar spindle and ensuring centrosome integrity during mitosis.^9^ However, its biological function in cancer remains to be elucidated.

In the last years, accumulating research has demonstrated that TROAP acts as a significant factor in the development and progression of various cancers.^10^ The upregulation of TROAP is considered to be associated with poor prognosis of cancer cohort, and its dysregulated expression can inhibit the proliferation and metastasis ability of tumor cells, hinting that the TROAP is closely associated with the malignant behavior of tumor.^11^, ^12^ For instance, Jing et al.^10^ revealed that TROAP is upregulated, resulting in a poor prognosis in gastric cancer patients. Through downregulated TROAP, the proliferation, cell cycle, invasion, and migration ability of gastric cancer cells could be inhibited. In addition, another study revealed that TROAP could mediate the cell cycle and facilitate the growth of glioma cells by regulating the Wnt/β–catenin pathway.^13^ Importantly, our previous study has found that the expression level of TROAP is correlated with the survival prognosis of STS.^14^ However, the prognostic value and specific functions of TROAP in STS are still undetermined. Therefore, it is necessary to comprehensively analyze the role of TROAP in STS.

Based on The Cancer Genome Atlas (TCGA), Gene expression Omnibus (GEO), and other tumor public databases, we explored the expression and prognosis significance of TROAP in STS. At the same time, a series of bioinformatics methods were carried out to investigate the relevance of TROAP and biological function, chemotherapy, and immunotherapy response of patients with STS. Also, experiments were conducted to confirm the role of TROAP in STS. We hope our study can further reveal the latent capacity of TROAP as a prognostic biological marker and its underlying molecular mechanism in STS and give a novel insight for the targeted therapy of STS.

## 2 RESULTS

### 2.1 TROAP is upregulated in a variety of tumors

**FIGURE 1:**
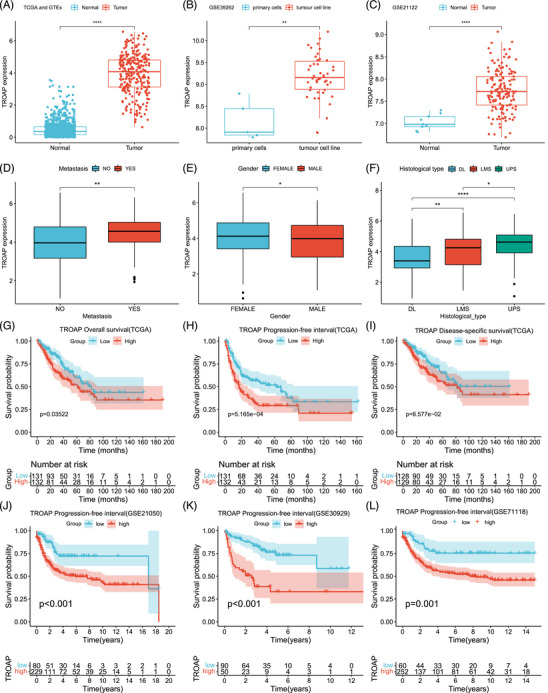
The expression, clinical significance, and survival analysis of TROAP in STS. (A) The TROAP expression in 263 STS tissue samples and 911 normal tissue samples. Statistical tests: Student's *t*‐test. (B) The TROAP expression of the primary cell line (*n* = 5) and tumor cell line (*n* = 46) in GSE39262 dataset. Statistical tests: Student's *t*‐test. (C) The TROAP expression of the normal tissue (*n* = 9) and tumor tissue (*n* = 149) in the GSE21122 dataset. Statistical tests: Student's *t*‐test. (D) Differences in the expression of TROAP between metastatic (*n* = 59) and nonmetastatic (*n* = 120) STS patients. Statistical tests: Student's *t*‐test. (E) Differences in the expression of TROAP between male (*n* = 119) and female (*n* = 144) STS patients. Statistical tests: Student's *t*‐test. (F) The TROAP expression among patients with different histological types (DL: *n* = 58; LMS: *n* = 105; UPS: *n* = 50). Statistical tests: Student's *t*‐test. (G) KM analysis of OS for high (*n* = 132) and low (*n* = 131) TROAP expression groups in TCGA cohorts. Statistical tests: Log‐rank test. (H) KM analysis of PFI for high (*n* = 132) and low (*n* = 131) TROAP expression groups in TCGA cohorts. Statistical tests: Log‐rank test. (I) KM analysis of DSS for high (*n* = 129) and low (*n* = 128) TROAP expression cohorts. Statistical tests: Log‐rank test. (J) KM analysis of PFI for high (*n* = 229) and low (*n* = 80) TROAP expression groups in the GSE21050 dataset. Statistical tests: Log‐rank test. (K) KM analysis of PFI for high (*n* = 50) and low (*n* = 90) TROAP expression groups in the GSE30929 dataset. Statistical tests: Log‐rank test. (L) KM analysis of PFI for high (*n* = 252) and low (*n* = 60) TROAP expression groups in the GSE71118 dataset. Statistical tests: Log‐rank test. KM, Kaplan–Meier; OS, overall survival; PFI, progression‐free interval; DSS, disease‐specific survival; DL, dedifferentiated liposarcoma; LMS, leiomyosarcomas; UPS, undifferentiated pleomorphic sarcoma. *p < 0.05, **p < 0.01, ***p < 0.001, ****p < 0.0001; ns, not significant.

Based on the TCGA database, we preliminary analyzed the expression of TROAP in pan‐cancer. We observed that TROAP was upregulated in most types of tumor tissues (Figure S1A). Again, the comparison between the cancer tissue and adjacent paired normal tissue shows the same results (Figure S1B). Considering that the number of tumor mutation burdens (TMB) and microsatellite instability (MSI) are essential indicators for predicting immune efficacy, we explored the association between TROAP, TMB, and MSI. We observed that TROAP is positively correlated with TMB in most tumors and negatively correlated with TMB in Thymoma (THYM) (Figure S1C). In addition, we also found a close relationship between TROAP and MSI in various tumors (Figure S1D). Subsequently, we further focused on investigating the expression of TROAP in STS. By combining tumor tissue data in the TCGA database with normal tissue data in the Genotype‐Tissue Expression (GTEx) database, our observation was that TROAP indicates a trend of significant upregulation in STS (Figure 1A). Consistently, the GSE39262 and GSE21122 datasets further confirm that TROAP exhibits an enhanced trend at both the tissue and cellular levels in STS (Figures 1B and C). Collectively, these above results suggest that TROAP is abnormally expressed in a variety of tumors, especially STS, implying that TROAP may play a unique role in STS.

### 2.2 The expression of TROAP could serve as an independent predictor for the prognosis of STS

Next, we compared the differences in TROAP expression between STS patients stratified according to different clinical features. The results indicate no difference in the TROAP expression in STS patients with different ages, margin status, and new tumor events (Figure S2). However, female and metastatic patients have a more augmented TROAP expression than male and non‐metastatic patients (Figures 1D and E). Meantime, the expression of TROAP is different under distinct histological conditions (Figure 1F). This hints that the TROAP may be associated with the clinical prognosis of STS. Therefore, we proceeded further to perform a survival analysis. As shown in Figures 1G–I, the STS patients with low TROAP expression have an enhanced survival rate compared with STS patients with high TROAP expression levels, whether overall survival (OS) or progression‐free interval (PFI). Disease‐specific survival (DSS) also exhibited the same trend, although not statistically significant. Additionally, we further confirmed the relevance between TROAP and the prognosis of STS using an external validation set. Excitingly, the external validation results are consistent with previous survival analyses exhibiting that the STS patient with a lower TROAP expression has an improved prognosis (Figures 1J–L). Taken together, these above results suggest that TROAP may play a carcinogenic role in STS, leading to poor prognosis in STS patients.

**FIGURE 2:**
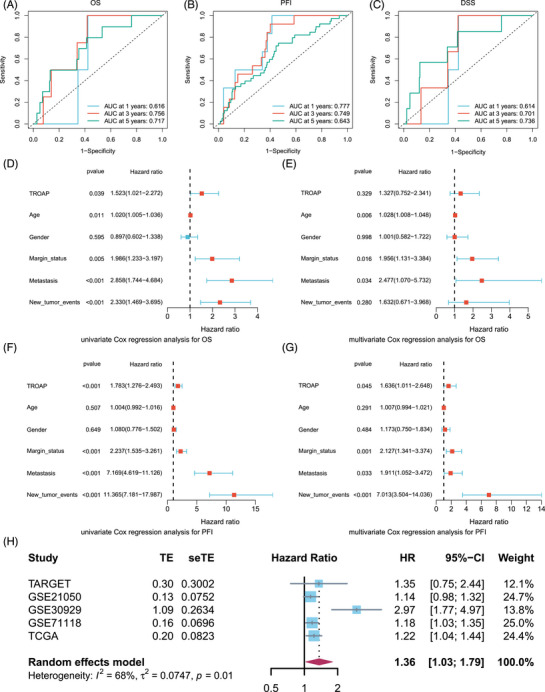
Prognostic prediction value of TROAP in STS. (A) ROC curves to predict the sensitivity and specificity of 1‐, 3‐, and 5‐year OS (*n* = 263) according to TROAP expression. (B) ROC curves to predict the sensitivity and specificity of 1‐, 3‐, and 5‐year PFI (*n* = 263) according to TROAP expression. (C) ROC curves to predict the sensitivity and specificity of 1‐, 3‐, and 5‐year DSS (*n* = 257) according to TROAP expression. (D and E) The univariate and multivariate Cox regression analysis for OS (*n* = 263) in the TCGA cohort. Statistical tests: Cox regression analysis. (F and G) The univariate and multivariate Cox regression analysis for PFI (*n* = 263) in the TCGA cohort. Statistical tests: Cox regression analysis. (H) The meta‐analysis of TROAP prognostic value was based on TCGA, TARGET, GSE21050, GSE30929, and GSE71118 (*n* = 1109). OS, overall survival; PFI, progression‐free interval; DSS, disease‐specific survival; AUC, area under curve.

Subsequently, we further drew the receiver operating characteristic (ROC) curve to evaluate the TROAP diagnostic value for predicting the prognosis of STS. For OS, the areas under 1‐, 3‐, and 5‐year ROC curves were 0.616, 0.756, and 0.717, respectively (Figure 2A). The areas under 1‐, 3‐, and 5‐year ROC curves for PFI were 0.777, 0.749, and 0.643 (Figure 2B). The areas under 1‐, 3‐, and 5‐year ROC curves for DSS were 0.614, 0.701, and 0.736, respectively (Figure 2C). Additionally, the univariate and multivariate analyses were also applied to evaluate the prediction performance. In predicting OS, the univariate analysis indicates that the hazard ratio (HR) value is 1.523 (*p* = 0.039, 95% confidence interval [95% CI] = 1.021–2.272), while the HR value of multivariate analysis shows no significant significance (Figures 2D and E). However, the univariate and multivariate analyses for predicting PFI are HR = 1.783 (*p* < 0.001, 95% CI = 1.276–2.493) and HR = 1.636 (*p* = 0.045, 95% CI = 1.011–2.648), respectively (Figures 2F and G). Consistently, meta‐analysis results also exhibit that the TROAP is an unfavorable factor for STS patients (*p* = 0.01, HR = 1.36, 95% CI = 1.03–1.79) (Figure 2H). Collectively, TROAP could serve as an independent risk assessment factor for the prognosis of STS, which has a robust performance.

### 2.3 The TROAP‐derived genomic signature exhibits robust performance for the prognosis prediction of STS

**FIGURE 3:**
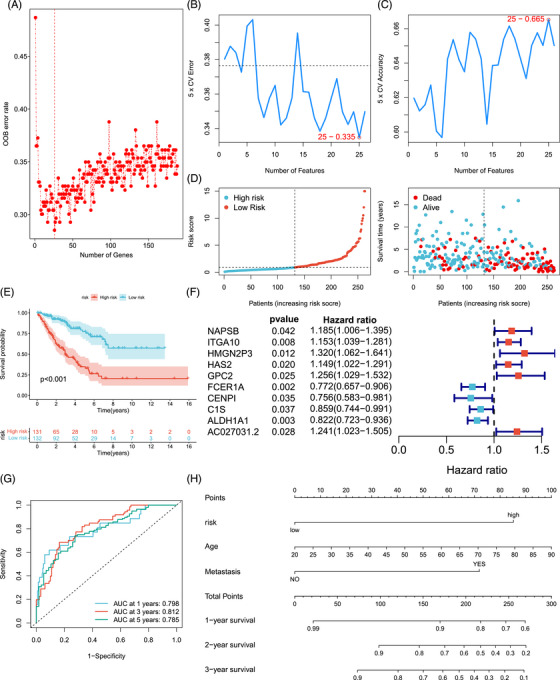
Construction of TROAP‐derived genomic signature in STS. (A) The important variables for STS were screened by OOB analysis. (B and C) The gene set with the highest accuracy was screened by the SVMRFE machine algorithm. (D) Ranked dots and scatter plots showing the TROAP‐derived risk score distribution and patient survival statuses (*n* = 263). (E) KM analysis of OS curves for the STS cohort with different TROAP‐derived risk scores (low risk:132, high risk: 131). Statistical tests: Log‐rank test. (F) The forest plots of ten TROAP‐derived signature genes with HR and 95% CI. Statistical tests: Cox regression analysis. (G) ROC curves to predict the sensitivity and specificity of 1‐, 3‐, and 5‐year OS (*n* = 263) according to TROAP‐derived risk score. (H) A prognostic nomogram consists of age, metastasis status, and TROAP‐derived risk scores for predicting the 1‐, 2‐, and 3‐year survival rates of STS patients (*n* = 263). HR, hazard ratio; 95% CI, 95% confidence interval; OOB, out‐of‐bag.

The generation and development of STS is a complex and complicated process, which often involves the participation of multiple genes. Therefore, we further constructed a TROAP‐derived genomic signature to predict the prognosis of STS accurately. Initially, we identified 852 differential expressed genes between the high‐ and low‐TROAP expression groups (Figure S3A), of which 641 were upregulated, and 211 were downregulated (Figure S3B). Through univariate COX regression screening, we then obtained 188 TROAP‐derived genes associated with the prognosis of STS (Table S1). Finally, the above genes were screened based on machine learning and multivariate regression analysis, resulting in a successfully constructed predictive model consisting of 10 TROAP‐derived genes (Figures 3A–C). Figure 3F displays the HRs a of these 10 TROAP‐derived genes. According to the risk score calculation formula, we calculated the TROAP‐derived risk score of each STS patient and divided the STS patients into high and low‐risk groups. We observed that the cohort with a higher risk score has more deaths (Figure 3D). Similarly, the STS patients with low TROAP‐derived risk scores have an enhanced survival prognosis (Figure 3E), and the areas under the ROC curve are 0.798 (1 year), 0.812 (3 years), and 0.785 (5 years) (Figure 3G).

Furthermore, we also developed a nomogram based on this model and the clinical characteristics of STS patients (Figure 3H), and the nomogram exhibits a preferable consistency with the actual observed value of STS (Figure S4). In sum, these above results imply that the TROAP‐derived signature has a superior predictive capacity.

### 2.4 The relationship between TROAP and tumor immune microenvironment

**FIGURE 4:**
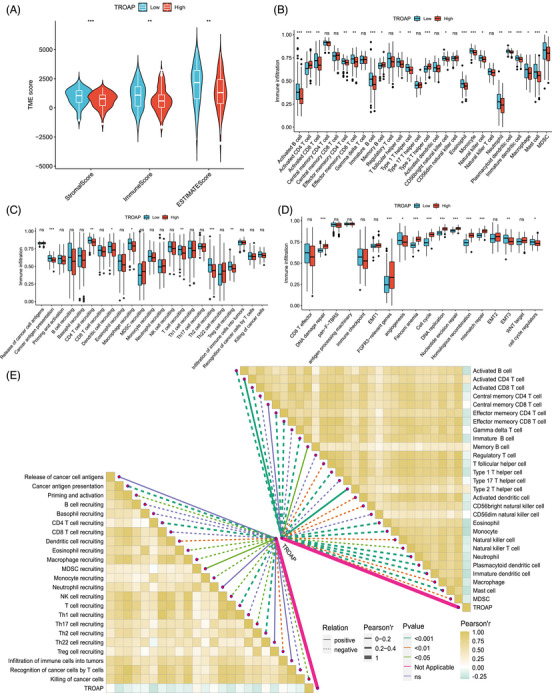
Association of TROAP with tumor immunity in STS. (A) The TME score difference between low (*n* = 131) and high (*n* = 132) TROAP expression subpopulations. Statistical tests: Student's t‐test. (B) The immune cells abundance between low (*n* = 131) and high (*n* = 132) TROAP expression subpopulations. Statistical tests: Student's *t*‐test. (C) The activities of the cancer immunity cycle in low (*n* = 131) and high (*n* = 132) TROAP expression subpopulations. Statistical tests: Student's *t*‐test. (D) The activation of known biological signatures in low (*n* = 131) and high (*n* = 132) TROAP expression subpopulations. Statistical tests: Student's *t*‐test. (E) Associations of TROAP expression with the activities of cancer immunity cycle and known biological signatures. The solid line represents positive correlation; the dotted line represents negative correlation; the thickness of the line and the color of the square represent the Pearson correlation coefficient, and the color of the line represents the *p* value. Statistical tests: Pearson correlation test. TME, tumor microenvironment; ns, not significant.

Based on the crucial role of the tumor immune microenvironment in malignancy progression and occurrence, the association between tumor immune infiltration and TROAP in STS was further investigated. Preliminarily, we observe that STS patients with low TROAP expression have enhanced stromal, immune, and estimate scores, implying that they may have an improved immune status (Figure 4A). Subsequently, the single‐sample gene set enrichment analysis (ssGSEA) results display that the most immune cells infiltrated degree is more abundant in the low TROAP expression group (Figure 4B). Meanwhile, the STS patient with a diminished TROAP expression exhibits an enhanced trend of several immune cells, such as recruiting CD4 T cells, dendritic cells, macrophages, and so on (Figure 4C). Also, we observe that the individuals with TROAP overexpression mainly focus on several vital pathway components, such as cell cycle, DNA replication, and so on (Figure 4D). Also, we revealed a significant negative association of TROAP with immune cell infiltration and immune‐related function (Figure 4E). Collectively, it can be preliminarily inferred that the TROAP expression change in tumor cells may result in the tumor immune status dysregulation, which may count for the poor prognosis of STS patients.

### 2.5 TROAP abnormal expression affects immunotherapy and chemotherapy

**FIGURE 5:**
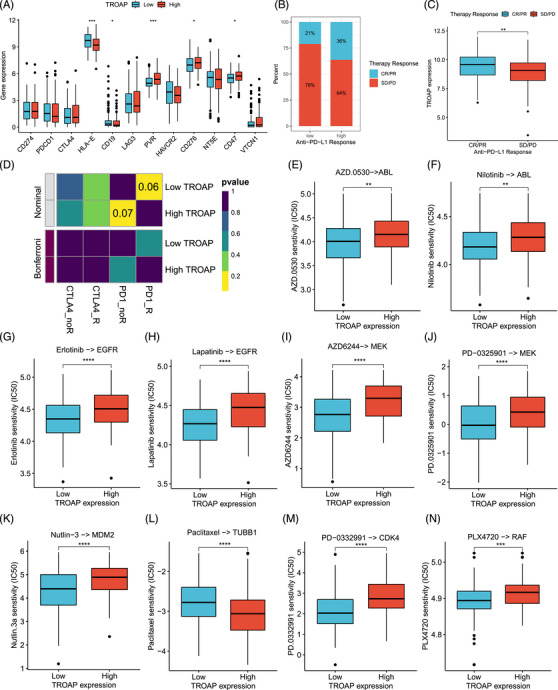
Association of TROAP expression with immunotherapy response and IC50 for different drugs. (A) Expression of immune checkpoints in the high (*n* = 132) and low (*n* = 131) TROAP expression groups. Statistical tests: Student's *t*‐test. (B) The difference in the percentage of CR/PR (*n* = 68) and SD/PD (*n* = 230) patients between distinct TROAP expression groups in the Imvigor210 dataset. (C) The difference in TROAP expression between CR/PR (*n* = 68) and SD/PD (*n* = 230) patients in the Imvigor210 dataset. Statistical tests: Student's *t*‐test. (D) The immunotherapy response of low and high TROAP expression subpopulations. Statistical tests: SubMap analysis. (E–N) The IC50 of AZD0530, nilotinib, erlotinib, lapatinib, AZD6244, PD‐0325901, nutlin‐3, paclitaxel, PD‐0332991, and PLX4720 between low (*n* = 131) and high (*n* = 132) TROAP expression subpopulations. Statistical tests: Wilcoxon rank sum test. SD, stable disease; PD, progressive disease; CR, complete response; PR, partial response; IC50, half maximal inhibitory concentration. *p < 0.05, **p < 0.01, ***p < 0.001, ****p < 0.0001; ns, not significant.

It is well known that immune checkpoints are closely related to the response to immunotherapy.^15^ Therefore, we analyzed the relationship between TROAP expression and immune checkpoints. We observed HLA‐E and CD19 exhibit an elevated trend, while PVR, CD276, and CD47 are reduced in the low TROAP expression group (Figure 5A). Meanwhile, we found that TROAP is positively correlated with PVR, CD276, and CD47 but negatively correlated with HLA‐E and CD19 (Figure S5). Considering the association of the immune checkpoints and TROAP, we further performed analysis to preliminarily assess whether TROAP could serve as an effective predictor for immunotherapy response. In the cohort that received immunotherapy (Imvigor210), the CR/PR and SD/PD proportions in the low TROAP expression group were 21 and 79%, respectively. In comparison, the CR/PR and SD/PD proportions in the high TROAP expression group were 36 and 64%, respectively (Figure 5B). Additionally, the expression level of TROAP was higher in the CR/PR population than in the SD/PD population (Figure 5C). However, we observed no significant difference in response to immunotherapy in STS patients with different TROAP expression levels (Figure 5D).

Moreover, drug therapy is another crucial means of tumor treatment.^16^ So, we evaluated the difference in the half maximal inhibitory concentration (IC50) of some commonly used targeted chemotherapy agents between the STS patients with different TROAP expressions to provide a potential reference for drug therapy selection. We found that the low TROAP expression group has significantly lower IC50 for AZD.0530, nilotinib, erlotinib, lapatinib, AZD6244, PD‐0325901, nutlin‐3, PD‐0332991, and PLX4720 compared with the high TROAP expression group (Figures 5E–K, M, and N). In comparison, the high TROAP expression group has a lower IC50 for paclitaxel (Figure 5L). Herein, the IC50 difference between distinct TROAP expression cohorts may help clinicians select rational drug and personalized treatment for STS.

### 2.6 TROAP is overexpressed in STS

**FIGURE 6:**
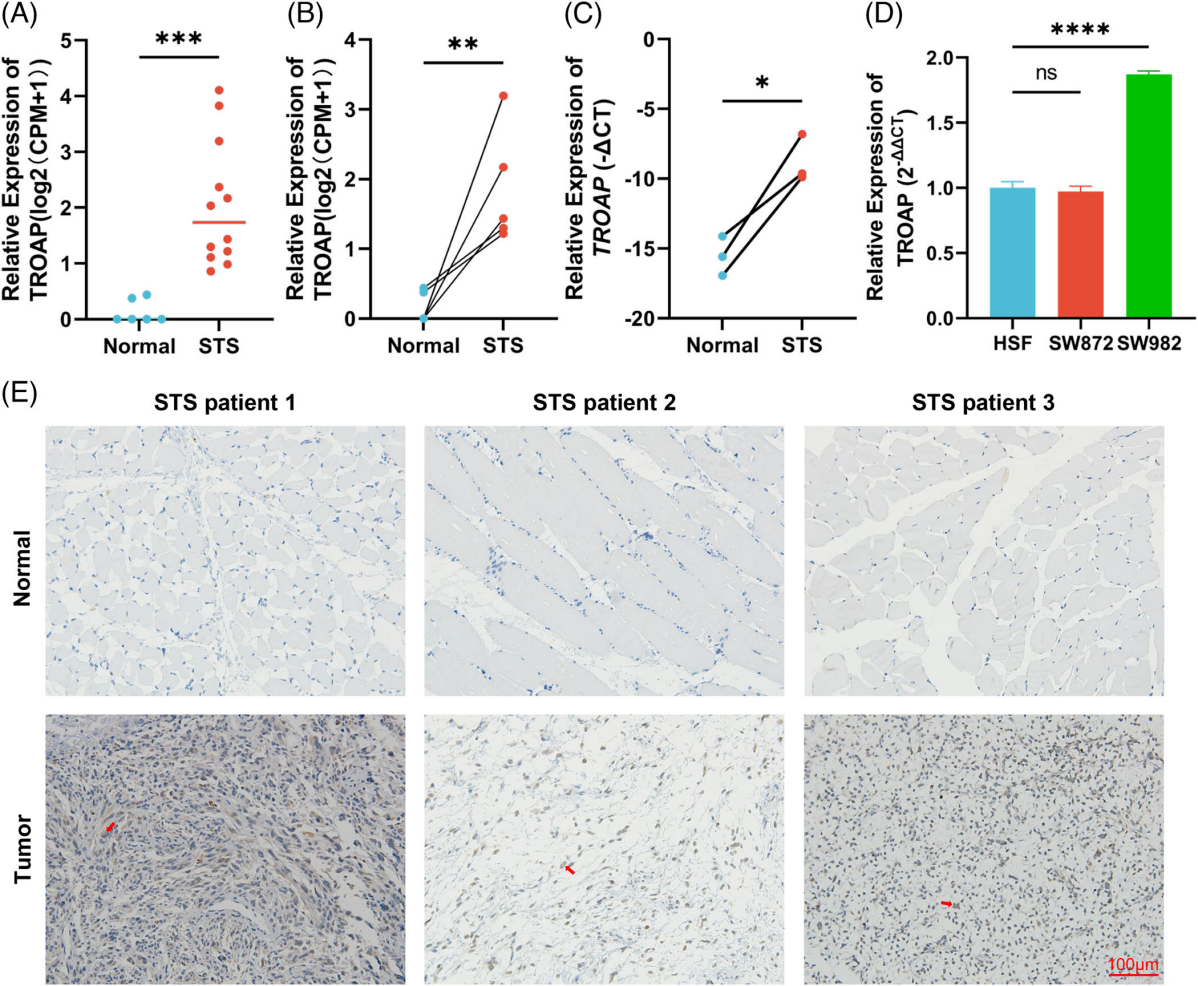
The TROAP was elevated in STS tissue and cell. (A) The TROAP expression level in sequencing data of 12 STS and six normal tissues. Statistical tests: Student's *t*‐test. (B) The TROAP expression level in sequencing data of six paired STS and normal tissues. Statistical tests: Student's *t*‐test. (C) The TROAP expression level in three paired STS tissues and normal tissues was detected by RT‐qPCR (*n* = 3 for each group). Statistical tests: Student's *t*‐test. (D) The expression of TROAP in HSF and SW982 cell lines was detected by RT‐qPCR (*n* = 3 for each group). Statistical tests: Student's *t*‐test. (E) Immunohistochemical staining of TROAP (1:500) in serial sections of paired tumors and normal tissue from three STS patients, red arrows demonstrate positive TROAP expression; scale bars, 100 μm. CPM, counts per million; STS, soft tissue sarcoma; HSF, human skin fibroblast cell line; SW982: the human synovial sarcoma cell line. *p < 0.05, **p < 0.01, ***p < 0.001, ****p < 0.0001; ns, not significant.

To confirm the reliability of our analysis, we further validated the expression level of TROAP at the tissue and cell levels. Our sequencing results (12 STS tissue and six normal tissue) display that the TROAP is significantly upregulated in STS tissues (Figure 6A). Meanwhile, the six paired tissue data in our sequencing expressions also indicate the same trend (Figure 6B). Additionally, we also found the enhanced expression of TROAP in STS tissue by polymerase chain reaction (PCR) detection (Figure 6C). Consistently, the expression level of TROAP is ameliorated in the STS cell line SW982 compared with human skin fibroblast (HSF), while no significant change is observed in SW872 (Figure 6D). Also, immunohistochemistry (IHC) represents positive staining for TROAP in STS tissues and negative staining for TROAP in normal muscle tissues, indicating the protein expression level of TROAP is elevated in STS (Figure 6E). Collectively, these results further confirmed the abnormal overexpression of TROAP in STS.

### 2.7 TROAP promotes the proliferation, migration, and invasion of STS cell

**FIGURE 7:**
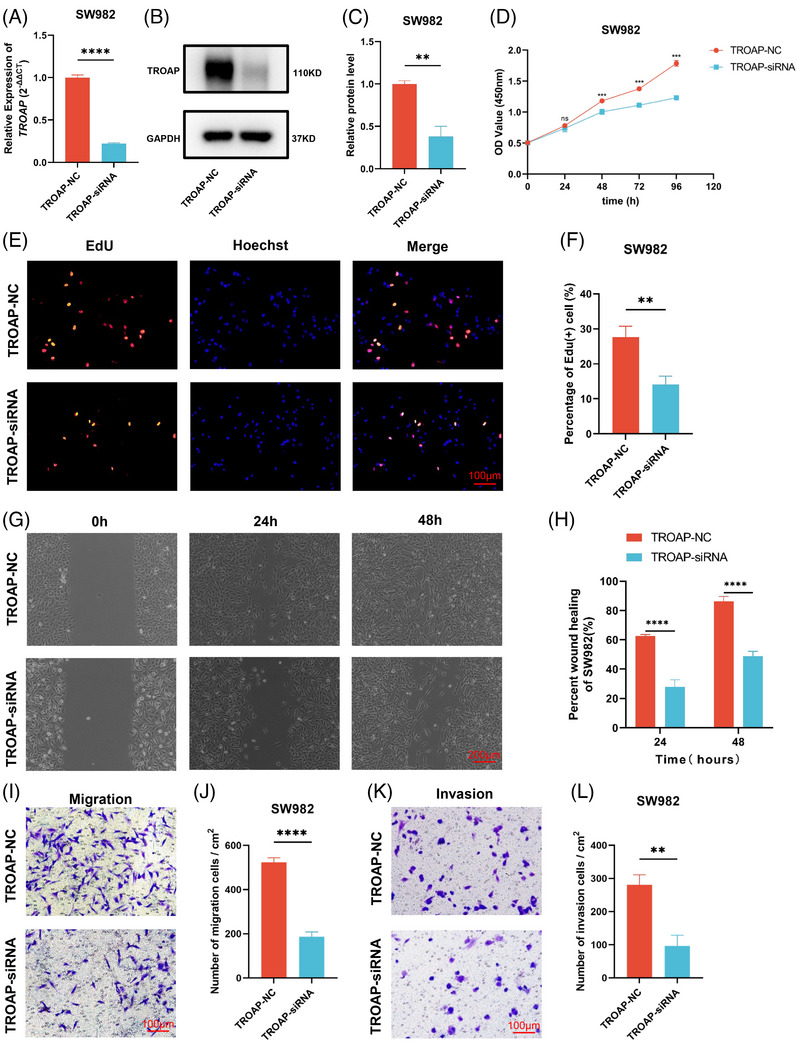
The proliferation, migration, and invasion abilities of the SW982 cells were inhibited with the downregulation of TROAP. (A) TROAP knockdown efficiency of siRNA in the SW982 cell line was detected by RT‐qPCR (*n* = 3 for each group). Statistical tests: Student's *t*‐test. Total RNA was extracted 48 h after transfection and used to detected mRNA levels. (B) TROAP knockdown efficiency of siRNA in the SW982 cell line was detected by western blot. Total protein was extracted 72 h after transfection and used to detected protein levels. (C) The quantification of TROAP protein levels was detected by western blot (*n* = 3 for each group). Statistical tests: Student's *t*‐test. The TROAP expression was knocked down by siRNA. (D) The growth curves of SW982 cells in NC and TROAP‐siRNA groups were examined by CCK‐8 assays (*n* = 3 for each group). The transfected SW982 was digested, counted, and then the cells were plated in a 96‐well plate. After that, adding CCK‐8 and detected the absorbance of the solution at 0, 24, 48, 72, and 96 h, respectively. Statistical tests: two‐way ANOVA. (E) The proliferation of SW982 cells in NC and TROAP‐siRNA groups were determined by EdU staining, and EdU incorporation was calculated as EdU (+) cells/total cells. The transfected SW982 cells were seeded in 12‐well plates and were stained as described in section Materials and Methods after 48 h of incubation. Scale bars, 100 μm. (F) The quantified result of Edu assay by Image J (*n* = 3 for each group). Statistical tests: Student's *t*‐test. (G) The migration capability of SW982 cells in NC and TROAP‐siRNA groups was determined by wound healing assay. When the SW982 cells grow to 90% confluence after transfected, scratched each well with a 200 μL sterile pipette tip and record the change of scratch after scratches 24 and 48 h. Scale bars, 200 μm. (H) The relative quantitative comparison of wound width of the wound healing assay (*n* = 3 for each group). Statistical tests: two‐way ANOVA. (I) The migration ability of SW982 cells in NC and TROAP‐siRNA groups were tested by transwell assay. Seed cells according to the section *Materials and Methods* and detect the number of cells passing through the chamber after 48 h. (J) The quantification of results from transwell migration assays (*n* = 3 for each group). Scale bars, 100 μm. Statistical tests: Student's *t*‐test. (K) The invasion ability of SW982 cells in NC and TROAP‐siRNA groups were tested by transwell assay. Seed cells according to the section *Materials and Methods* and detect the number of cells passing through the chamber after 48 h. (L) The quantification of results from transwell invasion assays (*n* = 3 for each group). Scale bars, 100 μm. Statistical tests: Student's *t*‐test. NC, negative control; siRNA, small interfering RNA. *p < 0.05, **p < 0.01, ***p < 0.001, ****p < 0.0001; ns, not significant.

Given the upregulation trend of TROAP in SW982, we selected this cell line for subsequent in vitro experimental verification to further investigate the impact of TROAP on the malignant biological behavior of STS. First, we used small interfering RNA (siRNA) to interfere with the TROAP expression in SW982. The results illustrate that the siRNA could significantly attenuate the expression of TROAP at both mRNA and protein levels (Figures 7A–C). Subsequently, we performed a cell counting kit‐8 (CCK‐8) assay to assess the proliferation ability of the SW982 cell line with different TROAP expressions. The result indicates that the optical density values of the SW982 in the TROAP‐siRNA group are significantly lower than those in the TROAP‐NC group at 48, 72, and 96 h (Figure 7D). The 5‐ethynyl‐2′‐deoxyuridine (EdU) staining is another method to detect the proliferation ability, and more EdU‐positive cells mean a stronger cell proliferation ability. Our result reveals that the mean EdU‐positive cells in the TROAP‐NC group and TROAP‐siRNA group are approximately 27.57 and 14.11%, respectively (Figures 7E and F). These results imply that reducing TROAP in SW982 could diminish its proliferation ability. Meanwhile, we also performed a wound healing assay and transwell assay to evaluate the effect of TROAP expression level on the migration and invasion ability of SW982. As present in Figures 7G and H, the wound healing assay indicates that the mean percentage wound healing of SW982 at 24 and 48 h in the TROAP‐NC group are 62.51 and 86.29%, while those in the TROAP‐siRNA group are 27.90 and 48.70%, respectively. Similarly, the mean amount of SW982 migration in the TROAP‐siRNA group (62.67) is less than that in the TROAP‐NC group (176) (Figures 7I and J). These suggest that the migration ability of SW982 is significantly reduced after TROAP is knocked down. Finally, the transwell invasion assay also displays that the mean number of SW982 invades the lower chamber in the TROAP‐NC and TROAP‐siRNA groups are 94.33 and 32.33, respectively (Figures 7K and L), hinting that the downregulation of TROAP could diminish the invasion ability of SW982. Hence, we can roughly infer that the downregulation of TROAP could significantly attenuate the proliferation, migration, and invasion ability of STS cells.

### 2.8 The potential function of TROAP in STS

**FIGURE 8:**
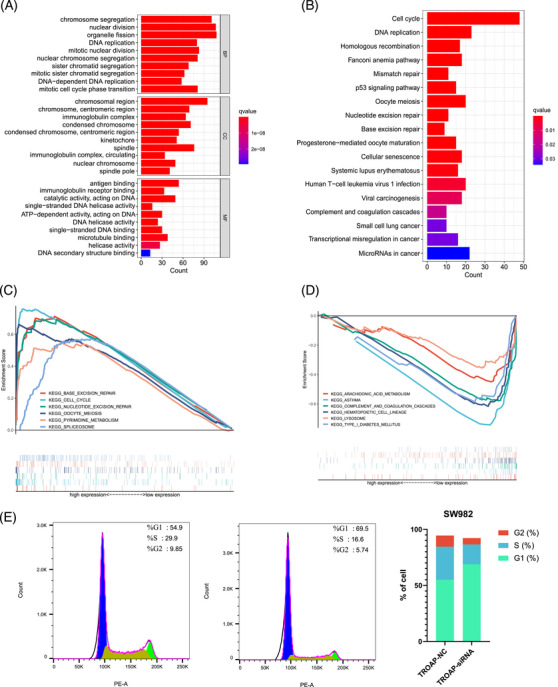
The potential biological function of TROAP in STS. (A) The results of GO enrichment analysis between the different TROAP expression cohorts (using c5.all.v5.1.symbols.gmt). (B) The KEGG pathway enrichment analysis result between the different TROAP expression cohorts (using c2.cp.kegg.v6.2.symbols.gmt). (C) GSEA for the signaling pathways activated in high TROAP expression cohort. (D) GSEA for the signaling pathways activated in low TROAP expression cohort. (E) The flow cytometric analysis results of the cell cycle for SW982 cells in NC and TROAP‐siRNA groups (*n* = 3 for each group). The transfected SW982 cells were collected and tested according to the section *Materials and Methods*. NC, negative control; siRNA, small interfering RNA. *p < 0.05, **p < 0.01, ***p < 0.001, ****p < 0.0001; ns, not significant.

Finally, we further explored the potential biological functions of TROAP in STS. Figures 8A and B illustrate the Gene Ontology (GO) and Kyoto Encyclopedia of Genes and Genomes (KEGG) results, and we found that the cell cycle is ranked first in the KEGG enrichment results. At the same time, GSEA results indicate that STS patients with low TROAP expression are mainly concentrated in arachidonic acid metabolism, asthma, complement, and coagulation cascades, hematopoietic cell linage, lysosome, type I diabetes mellitus (Figure 8C), for STS patients with high TROAP expression, base excision repair, cell cycle, nucleotide excision repair, oocyte meiosis, pyrimidine metabolism, and spliceosome are their main enriched pathways (Figure 8D). Considering the KEGG and GSEA results both indicating that STS patients with TROAP overexpression are enriched in the cell cycle, we further performed flow cytometry detection to explore whether the TROAP expression could affect the cell cycle of SW982. The results reveal that the percentage of cells in the G1 phase increases (TROAP‐NC: 55.07%, TROAP‐siRNA: 68.83%), while the percentage of cells in the S phase (TROAP‐NC: 29.50%, TROAP‐siRNA: 17.67%) and G2 phase (TROAP‐NC: 9.923%, TROAP‐siRNA: 5.75%) decreases after disturbing the expression level of TROAP in SW982 (Figure 8E). Hence, the above evidence distinguished that TROAP could arrest the cell cycle of STS cells.

## 3 DISCUSSION

STS is a rare, heterogeneous group of tumors exhibiting distinct mesenchymal differentiation with over 80 histologic subtypes.^17^ Despite STS accounts for less than 1% of all cancers, its incidence is the highest among rare malignancies.^18^ STS varies in origin, histology, and genetic markers, but a common feature is that patients in the advanced stage are often related to poor prognoses. Even though the treatment strategies have been implemented and continuously optimized, approximately one‐third of STS patients still die due to the disease.^19^ Therefore, early diagnosis, exploration of valuable prognostic indicators, and precise treatment are of great significance in improving the clinical prognosis of patients with STS. At present, several studies have proven that the TROAP is elevated in glioma, liver cancer, prostate cancer, colon cancer, and other tumor tissues, and its expression level is negatively correlated with the survival prognosis of tumor patients.^12^, ^13^, ^20^, ^21^ For instance, Gao et al.^22^ demonstrated that TROAP is overexpressed in clear cell renal cell carcinoma (ccRCC), which could accelerate the proliferation, migration, and invasion potentials of ccRCC cells. Until now, no in‐depth study has focused on the relationship between TROAP and the clinical prognosis and molecular mechanism of STS. Herein, we comprehensively explored the molecular characteristics and prognostic value of TROAP in STS through bioinformatics analysis and revealed its relationship with the immune microenvironment and chemotherapy, implying TROAP could serve as a biomarker for prognosis assessment and individualized, precise treatment of patients with STS. Also, we confirmed that TROAP is abnormally elevated in STS cells and tissue and could regulate the proliferation, migration, invasion, and cell cycle of STS cells to affect the malignant biological behavior of STS, which provides a new perspective for the study of the pathogenesis of STS.

Initially, we analyzed the expression of TROAP in various tumors based on the TCGA database, and the results illustrate that TROAP is overexpressed in multiple tumors. Further differential analysis based on STS tissue data in TCGA and normal tissue data in GTEx also indicates that TROAP is abnormally increased in STS. Additionally, consistent results are observed in the GEO validation result. Subsequently, we used survival analysis, ROC curve, single multivariate analysis, and meta‐analysis revealed that TROAP could be used as an independent prognostic factor for patients with STS and its expression level negatively correlative with the survival time of STS. Hence, the above results hint that detecting the expression level of TROAP may help predict the prognosis of STS.

It is well known that the prognosis of cancer is often related to multiple risk factors. A nomogram is a powerful tool for quantifying an individual's risk in a clinical setting by integrating various risk factors.^23^, ^24^ Therefore, we used the nomogram to predict 1‐, 3‐, and 5‐year OS probabilities by combining patient age, metastatic status, and a TROAP‐derived signature. Importantly, ROC curves also indicate the robust efficiency of the nomogram in predicting the clinical outcome of STS individuals. Moreover, the calibration curve confirms that the actual survival time is consistent with the estimated survival time using the nomogram. Therefore, the above results provide fresh insights for us to predict STS patients' prognosis accurately.

Although immunotherapy has presented excellent effects in treating some solid cancer, such as melanoma and bladder cancer,^25^, ^26^, ^27^ its clinical results are still unsatisfactory in more types of multiple tumors, including STS.^28^ One important reason involves our understanding of the interaction between tumor and immune status in STS is insufficient, and there is a lack of new diagnostic and treatment prediction targets. Generally, tumor immune cell infiltration is recognized as an independent predictor of tumor immune status and survival.^29^ Consequently, we further investigated the correlation between TROAP and tumor immune infiltration, trying to provide a new idea for explaining why TROAP affects the prognosis of STS. The results display that the enhanced expression of TROAP is associated with attenuated immune cell infiltration, decreased stromal and immune activation, and diminished activity of the cancer immune cycle, which may be accounting for the poor prognosis of STS with TROAP overexpression. Furthermore, we also observed an association between TROAP and many immune checkpoints expression, which motivated us to further assess the potential of TROAP as a predictor of response to immunotherapy.

Although the current results indicate no significant difference in the effect of immunosuppressants in STS patients with different TROAP expressions, this may be due to the lack of more cohort data on immunotherapy for STS, and a larger STS cohort in the future is needed for further validation. Tumor drug resistance is a significant problem that limits the efficacy of cancer chemotherapy drugs at present.^30^ Many cancer patients have substantial curative effects at the early stage of chemotherapy. However, with the extension of treatment duration, the drug resistance of cancer cells is enhanced, ultimately leading to treatment failure.^31^ The process of drug reutilization can provide new insights into the pathogenesis of disease and find new opportunities for drug intervention.^32^ Therefore, we analyzed the correlation between TROAP and preclinical and clinical drugs to discover potential treatments for STS. We found that TROAP is negatively associated with the sensitivities of AZD.0530, nilotinib, erlotinib, lapatinib, AZD6244, PD‐0325901, nutlin‐3, PD‐0332991, and PLX4720, which provides a potential drug for the treatment of patients with the low expression level of TROAP.

Moreover, we further verified the expression of TROAP in STS and its relationship using in vitro experiments. Our sequencing and PCR results show that the expression of TROAP is elevated in STS compared with normal tissues and cell lines. Next, we found that the proliferation, migration, and invasion abilities of STS cell lines are all inhibited after the TROAP expression has interfered with the siRNA. In agreement, Li et al.^20^ found that TROAP could directly bind DYRK1A and DYRK1B to form a protein complex, resulting in the retention of DYRK1A and DYRK1B in the cytoplasm. Subsequently, DYRK1 in the cytoplasm activates Akt/GSK‐3β signaling, enhancing the stability and nuclear localization of cyclin D1, thereby accelerating cell cycle progression and facilitating the malignant proliferation of HCC.^20^ Jin et al.^33^ demonstrated that TROAP, as a downstream regulator of EZH2, can activate the TWIST/c‐Myc pathway to regulate the progression of prostate cancer. Also, we observed that KEGG, GSEA, and flow cytometry results all exhibit that TROAP can interfere with the cell cycle of STS. The dysregulation of the cell cycle is an important hallmark of cancer.^34^ As previously reported, TROAP could affect the tumor progression and cell cycle of glioma cells via the Wnt/β‐Catenin signaling pathway.^13^ In prostate cancer, TROAP also takes part in regulating WNT3/surviving signaling pathways to affect cell progression.^12^ Based on the above research reports, we found that the Wnt signaling pathway is an important downstream signaling pathway of TROAP, hinting that TROAP might regulate this signaling pathway to affect the malignant biological behavior and cell cycle of STS cells. However, this is only our conjecture, the specific molecule mechanism still needs further exploration.

Despite several important discoveries in our research, some limitations remain inevitable. Initially, our study investigated the prognostic significance of TROAP for STS based on public datasets, more clinical cohorts need to be collected for further confirmation of the predictive performance in the future. Meanwhile, the effect of TROAP on STS has only been verified in vitro experiments, while in vivo animal experiments and mechanism exploration will be our team's future research focus. Finally, the specific mechanism of the TROAP expression change in tumor cells that causes the tumor immune microenvironment status, the change of drug sensitivity, and the possible molecular mechanism in drug sensitivity change need further clarification in future research.

## 4 CONCLUSION

In brief, our study comprehensively analyzed the molecular profile, oncogenic role, and immune and pharmacogenomic features of TROAP in STS. These results first time reveal that the upregulation of TROAP is relevant to the prognosis of STS and could promote the proliferation, migration, invasion, and cell cycle of STS cells, which may bring a new perspective to the treatment of STS.

## 5 MATERIALS AND METHODS

### 5.1 Data collection

The RNA‐Seq data were downloaded through the TCGA database (https://portal.gdc.cancer.gov/).^35^ The downloaded transcriptome data were integrated, and the ensemble ID was converted into gene names. After eliminating patients without survival information, a total of 263 STS patients were enrolled for further analysis. The gene expression profiles of 911 normal tissues (including 396 muscle tissues and 515 adipose tissues) in the GTEx (https://www.gtexportal.org/ home/) database were extracted and served as normal control tissue.^36^ In addition, the GSE39262 dataset (including five normal cell lines and 46 sarcoma cell lines) and the GSE21122 dataset (including nine normal tissues and 149 tumor tissues) were downloaded from The GEO database (https://www.ncbi.nlm.nih.gov/geo/) as external datasets for the TROAP expression verification.^37^ Moreover, the GSE21050 (including 309 STS patients), GSE30929 (including 140 STS patients), and GSE71118 (including 312 STS patients) were applied to validate the association of TROAP with the prognosis of STS. Moreover, the Imvigor210 dataset containing 298 urothelial carcinoma individuals accepted immunotherapy was also downloaded from the GEO database for subsequent analysis.

### 5.2 Immune infiltration analysis

For an exploration of the association between the TROAP expression and the immune microenvironment, various analysis was performed. First, the tumor microenvironment (TME) score was computed through the ESTIMATE algorithm.^38^ Besides, the ssGSEA algorithm was applied to explore the tumor‐infiltrating abundance of immune cells.^39^ The activities of all cancer immunity cycle steps and gene sets of known biological processes (BPs) obtained from previous researches were quantitative by the ssGSEA algorithm. The correlation between the TROAP expression level and these processes was also confirmed. Finally, the immune checkpoints were extracted from previous studies to explore the relationship between TROAP and immune checkpoints.^40^, ^41^, ^42^, ^43^

### 5.3 Differences in immunotherapy response

To help guide the treatment selection of STS, we used the subclass mapping (SubMap) algorithm to predict the response to immunotherapy inhibitors (anti‐CTAL‐4 and anti‐PD‐L1) in STS patients with high and low TROAP expression.^44^ The *p* value has been corrected by Bonferroni correction, and the patient is considered to have a response to immunotherapy when the Bonferroni *p* value is less than 0.05.

### 5.4 Drug sensitivity estimation

Based on the TROAP expression profiles from TCGA and the cell line expression profiles from the most extensive public pharmacogenomics database, Genomics of Cancer Drug Sensitivity (https://www.cancerrxgene.org/), we used the “pRRophetic” R package to construct a ridge regression model to calculate the IC50 of STS patients for chemotherapy drugs. We used the Wilcoxon sign‐rank test to compare the IC50 of chemotherapy agents between the STS patients with different TROAP expression.^45^, ^46^ All parameters are default values, and mean values replace duplicate gene expressions.

### 5.5 Generation of TROAP‐derived genomic model

To construct a TROAP‐derived prognosis evaluation model, we first categorized STS patients into low and high TROAP expression groups. Then, differential analysis was performed based on the “limma” package to screen the differential genes between the high and low TROAP expression groups, where the screening criteria were set as |log2FoldChange > 0.585| and the adjusted *p* values were <0.05.^47^ After identifying the differential genes, we further carried out univariate regression analysis to screen the genes relevant to prognosis. TROAP‐relevant genes with *p* value < 0.05 were determined as prognostic factors, which were input into the out‐of‐bag data (OOB) algorithm to obtain essential variables with the minimum value of OOB. Finally, the gene set with the highest accuracy was confirmed by the SVMRFE machine algorithm, and a prognostic model was constructed using multivariate Cox regression analysis.^40^ The TROAP‐relevant risk score was calculated according to the expression and regression coefficient of the model gene. The STS individuals were further divided into low and high‐risk groups based on the median risk score, which was convenient for subsequent analysis.

### 5.6 Functional annotation enrichment analysis of TROAP

To further understand the gene enrichment in STS, we conducted GO and KEGG pathway enrichment analysis given TROAP‐related genes using the clusterProfiler package.^48^ Among them, GO categories include BP, cellular component, and molecular function. Additionally, we also performed GSEA analysis using “c2.cp.kegg.v6.2.symbols.gmt” as a reference to investigate the activation of signaling pathways differences between STS cohorts with distinct TROAP expression.^49^

### 5.7 Sources of clinical specimens

The study was approved by the ethical committee of the Second Xiangya Hospital of Central South University. A total of 15 STS tissues and nine standard control tissues sample obtained from the Second Xiangya Hospital of Central South University were included in this study. Immediately after the tissue was isolated, it was rinsed with saline to minimize residual blood tissue on the surface of the tissue. Among them, three pairs of STS tissues and normal control tissues were used for quantitative real‐time PCR (RT‐qPCR) and IHC, and the remaining samples were used for sequencing.

### 5.8 Transcriptome analysis

As described in previous research, the tissue samples were sent to (Biomarker Technologies Ltd) for a full‐length transcriptome analysis.^50^ The BMKCloud analysis platform was applied for subsequent analysis according to the reference sequences and nanopore transcriptome sequencing data.

### 5.9 Cell lines and cell culture

As reported in the previous article,^51^ the human synovial sarcoma cell line (SW982) and liposarcoma cell line (SW872) obtained from American Type Culture Collection (ATCC) was selected as the experiment cell. In contrast, the HSF cell line from the Fenghui Biotechnology Co., Ltd was used as a normal control cell. The above cells were all cultured in Dulbecco's Modified Eagle Mproedium (DMEM; Procell) containing 10% fetal bovine serum (FBS; Procell) and 1% penicillin–streptomycin solution (Bioss), the culture temperature was 37°C, and the environment and humidity were 95% oxygen, 5% carbon dioxide, and 95% relative humidity, respectively.

### 5.10 Quantitative real‐time PCR

The cellular RNA was extracted following the procedures described in previous studies.^51^ While the RNA of normal and STS tissue was extracted using Trizol (Invitrogen). After rinsing the tissue, add 200 μL chloroform (Sigma) and leave for 15 min on ice. Then centrifuge (12,000×*g*, 4°C, 15 min) and transfer the RNA from the upper aqueous layer to a new Eppendorf (EP) tube. Add 100 μL of prechilled isopropanol (Sangon Biotech) to the EP tube, mix well, and then centrifuge again (12,000×*g*, 4°C, 10 min). To remove impurities, wash the RNA twice with 75% ethanol (Sangon Biotech) and then dissolve the RNA pellet with about 50 μL of RNase‐free pure water. Finally, RNA reverses transcription and RT‐qPCR were performed according to the previously described steps.^24^ Usually, glyceraldehyde 3‐phosphate dehydrogenase (GAPDH) is selected as an internal reference, and the relative expression level of TROAP is calculated by the 2^^−ΔΔCT^ method. Table S2 exhibits the primer sequences.

### 5.11 Cell transfection

The siRNA was purchased from Syngenbio, and the sequence of siRNA is listed in Table S2. The cells were plated at an appropriate cell density on the day before the transfection experiment, and the transfection was performed when the cell was 30−50% confluence. Prepare two enzyme‐free sterile EP tubes, add 250 μL Opti‐MEM (Gibco) and 5 μL siRNA (20  μM) to one of them, and add 250 μL Opti‐MEM and 5 μL lipo2000 (Invitrogen) to the other tube. Next, mix the above two and stand at room temperature for 15 min; add the solution to the corresponding cells and shake gently to distribute the complex evenly. Cells were cultured statically at 37°C, replaced with a complete medium after 6−8 h, and cultured appropriately for subsequent experiments.

### 5.12 Cell counting kit‐8

The CCK‐8 form New Cell & Molecular Biotech was applied to assess cell proliferation ability. The transfected SW982 was digested and counted, and then the cells were plated in a 96‐well plate (2000 cells/well). Next, add 100 μL serum medium containing 10 μL CCK‐8 to each well at 0, 24, 48, 72, and 96 h, respectively. After incubation at 37°C for 2 h, the absorbance of the solution was measured at 450 nm with a spectrophotometer.

### 5.13 EdU assay

The EdU Cell Proliferation Detection Kit (RiboBio) was used to determine cell proliferation ability. The transfected SW982 cells were seeded in 12‐well plates. After 48 h of incubation, cells were cultured using 50 μm EdU reagent (diluted with DMEM containing 10% FBS at 1:1000) for 2 h at 37°C. Then, fixed with 4% PFA and stained with Hoechest solution (diluted with DMEM containing 10% FBS at 1:100). Finally, observe and collect images under a fluorescence microscope.

### 5.14 Wound healing assay

The SW982 cells were plated at a suitable density in a six‐well plate for culture. When the cells grow to 90% confluence after transfected, scratched each well with a 200 μL sterile pipette tip. To remove the separation, washed the cell three times with 1×PBS. Then, 2 mL of the fresh serum‐free medium was added to each well and placed in a 37°C incubator for 24 and 48 h. Meanwhile, took pictures of the scratches with a phase‐contrast microscope after scratches 24 and 48 h.

### 5.15 Transwell assay

The transwell cell culture chamber was obtained from Corning. For migration ability assays, 200 μL of serum‐free medium containing 1 × 10^4^ transfected cells were inoculated into the upper chamber. On the other hand, 50 μL Matrigel (Corning; diluted using DMEM containing 10% FBS at 1:8) was loaded in a 24‐well chamber before cell inoculation, and the number of cell inoculation was changed to 2 × 10^4^ were applied for transwell invasion ability assays. Meantime, 600 μL of complete medium with 10% FBS was added to the lower chamber. After incubation for 48 h, the cells on the surface of the upper chamber were removed by cotton swabs. The upper chamber was fixed with 4% paraformaldehyde for 30 min at room temperature and stained with 1% crystal violet for 5 min. Finally, observe and record the staining results under a microscope.

### 5.16 Immunohistochemistry

IHC staining was performed as described in previous studies.^52^ Sections were incubated with primary antibody (TROAP; Proteintech; 13634‐1‐AP) in PBS containing 3% BSA overnight at 4°C, followed by secondary antibody incubation for 1 h at room temperature.

### 5.17 Western blot

The conduction of western blot was consistent with the previously described.^28^ The proteins in the cell were extracted with RIPA lysis buffer (New Cell & Molecular Biotech) containing proteinase and phosphatase inhibitors (New Cell & Molecular Biotech). The following antibodies were utilized: anti‐TROAP (Proteintech; 13634‐1‐AP), anti‐GAPDH (Affinity; T0004), anti‐rabbit IgG (Cell Signaling Technology; 7074), and anti‐mouse IgG (Zen Bioscience; 550108).

### 5.18 Flow cytometry

The Cell Cycle Staining Kit (MULTI SCIENCES) was used for flow cytometry detection. According to the operating manual, a proper amount of transfected SW982 cells were collected, and the supernatant was discarded by centrifugation. The cells were washed once with PBS, and the supernatant was discarded by centrifugation. After adding 1 mL DNA staining solution and 10 μL permeabilization solution, vortex for 5–10 s to mix, incubate at room temperature for 30 min in the dark. Finally, the samples were tested on a flow cytometer.

### 5.19 Statistical analysis

The R software (version 4.0.1) and GraphPad Prism (version 9.0.0) were applied for statistical analysis. Quantitative data are displayed in terms of mean ± SD. The differences between continuous variables were determined using Student's *t*‐test, analysis of variance (ANOVA), and Wilcoxon rank sum test. The Fisher Exact test was applied to compare unordered categorical variables. The R package “survival” was utilized for Kaplan–Meier (KM) analysis, and the log‐rank test was utilized to analyze the KM curves. The R package was used to plot ROC curves. Prognostic factors were identified by Cox regression analysis. Correlation coefficients were calculated by Spearman correlation analysis. The difference was considered statistically significant when the *p* value was less than 0.05.

## AUTHOR CONTRIBUTION

*Project design*: Shasha He and Zhihong Li. *Experimental methods*: Binfeng Liu, Chao Tu, Chenbei Li, Chengyao Feng, Hua Wang, and Haixia Zhang. *Data analysis*: Binfeng Liu and Chao Tu. *Manuscript writing*: Binfeng Liu and Chao Tu. All authors revised the manuscript and approved the final version.

## CONFLICT OF INTEREST STATEMENT

The authors declare no conflict of interest.

## ETHICS STATEMENT

The study received ethical approval from the Medical Ethics Committee of Second Xiangya Hospital of Central South University and all participants provided written informed consent (approval ID: 2022040).

## Supporting information

Supporting Information

## Data Availability

The data that support the findings of this study are available from the corresponding author upon reasonable request.
