# Supplementary material for: Integrative analysis of TROAP with molecular features, carcinogenesis, and related immune and pharmacogenomic characteristics in soft tissue sarcoma
Source: MedComm (2020). 2023 Sep 18;4(5):e369. doi: 10.1002/mco2.369 (PMC10507284; doi:10.1002/mco2.369)
Supplement: Supplementary file 1 — Supporting Information [file MCO2-4-e369-s001.docx]

**Integrative analysis of TROAP with molecular features, carcinogenesis and related immune and pharmacogenomic characteristics in soft tissue sarcoma.**

**Running title: TROAP in soft tissue sarcoma**

Chao Tu^1,2#^, Binfeng Liu^1,2#^, Chenbei Li^1,2^, Chengyao Feng^1,2^, Hua Wang^1,2^, Haixia Zhang^3^, Shasha He^3*^, Zhihong Li^1,2*^

^1^ Department of Orthopaedics, The Second Xiangya Hospital of Central South University, Changsha 410011, Hunan, China.

^2^ Hunan Key Laboratory of Tumor Models and Individualized Medicine, The Second Xiangya Hospital of Central South University, Changsha 410011, Hunan, China.

^3^ Department of Oncology, The Second Xiangya Hospital of Central South University, Changsha 410011, Hunan, China.

^#^These authors have contributed equally to this work and share the first authorship.

*Corresponding Authors:

Shasha He, MD, Department of Oncology, The Second Xiangya Hospital, Central South University, Changsha 410011, Hunan, China. email: heshasha611@csu.edu.cn.
Zhihong Li, MD, Department of Orthopaedics, The Second Xiangya Hospital, Central South University, Changsha 410011, Hunan, China. Email: lizhihong@csu.edu.cn.

**Supplementary Figures**





**Figure S1.** Analysis of expression, tumor mutational burden (TMB), and microsatellite instability (MSI) of TROAP across pan-cancer. A. The differential expression of TROAP in various cancers and corresponding normal tissue. B. comparison of the expression of TROAP between paired tumor and normal tissues. C. Radar chart shows the association of TROAP with TMB in each cancer type. D. Radar chart shows the association of TROAP with MSI across pan-cancer. ACC: adrenocortical carcinoma; BLCA: bladder urothelial carcinoma; BRCA: breast invasive carcinoma; CESC: cervical squamous cell carcinoma and endocervical adenocarcinoma; CHOL: cholangiocarcinoma; COAD: colon adenocarcinoma; DLBC: lymphoid neoplasm diffuse large B-cell lymphoma; ESCA: esophageal carcinoma; GBM: glioblastoma multiforme; HNSC: head and neck squamous cell carcinoma; KICH: kidney chromophobe; KIRC: kidney renal clear cell carcinoma; KIRP: kidney renal papillary cell carcinoma; LAML: acute myeloid leukemia; LGG: lower-grade glioma; LIHC: liver hepatocellular carcinoma; LUAD: lung adenocarcinoma; LUSC: lung squamous cell carcinoma; MESO: mesothelioma; OV: ovarian serous cystadenocarcinoma; PAAD: pancreatic adenocarcinoma; PCPG: pheochromocytoma and paraganglioma; PRAD: prostate adenocarcinoma; READ: rectum adenocarcinoma; SARC: sarcoma; SKCM: skin cutaneous melanoma; STAD: stomach adenocarcinoma; TGCT: testicular germ cell tumors; THCA: thyroid carcinoma; THYM: thymoma; UCEC: uterine corpus endometrial carcinoma; UCS: uterine carcinosarcoma; UVM: uveal melanoma. *P < 0.05, **P < 0.01, ***P < 0.001, ****P < 0.0001.



**Figure S2.** The TROAP expression among patients with different ages, margin status, and new tumor events.


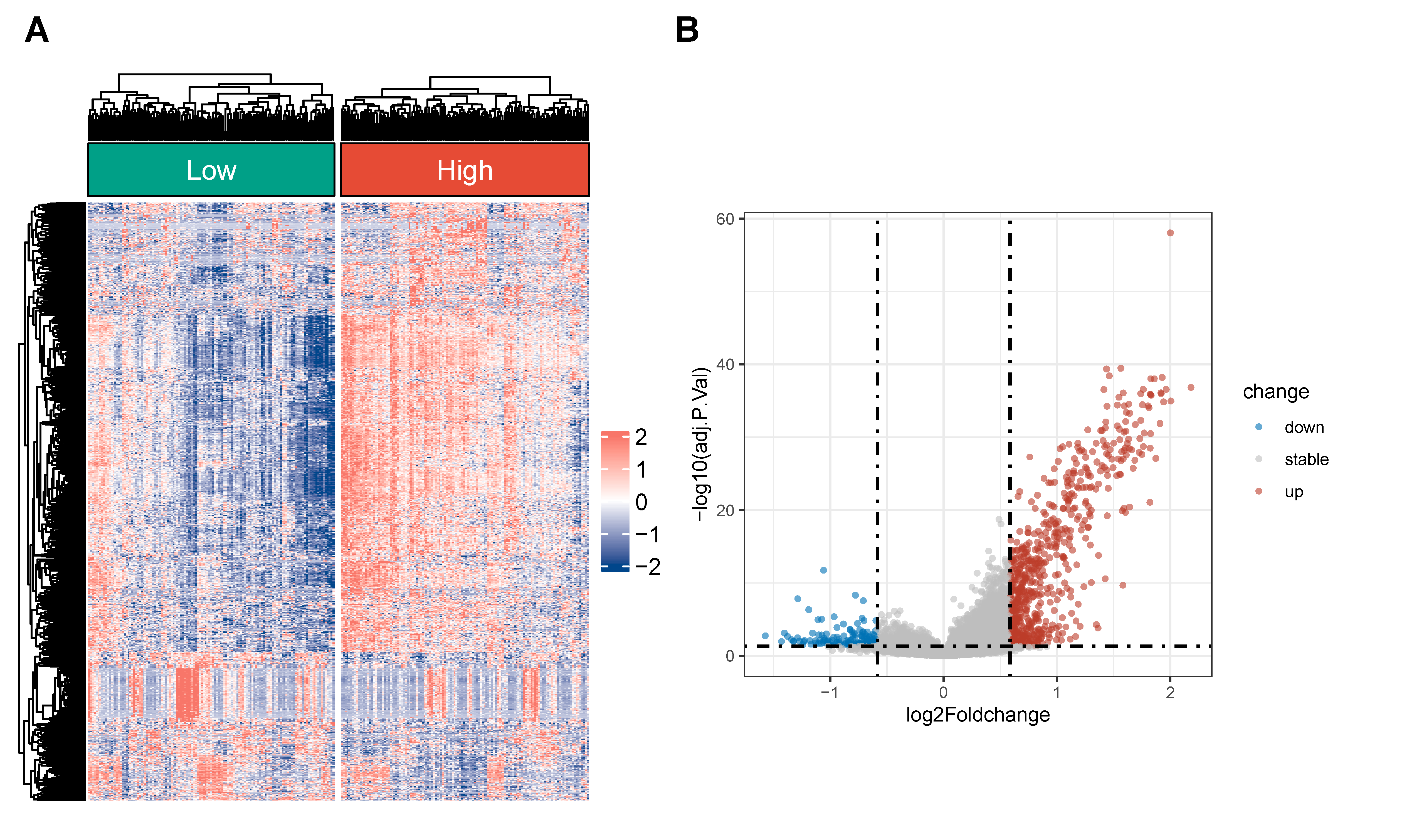


**Figure S3.** The differential expressed genes between the TROAP high and low expression groups. A. The heatmap of differentially expressed genes between the distinct TROAP expression groups. A. The volcano map of differentially expressed genes between the distinct TROAP expression groups.



**Figure S4.** The expression heat map of signature genes in STS and the predictive efficacy of nomogram. A. The heatmap of TROAP-derived signature gene in STS. B. The ROC curves at 1-, 3-, and 5-year survival predict by the nomogram. C-E. Calibration plots show the association of predicted 1-, 3-, and 5-year OS with actual survival duration.





**Figure S5.** The correlation between TROAP and immune checkpoint in STS patients.

**Supplementary Tables**

| **Table S1**. The univariate Cox regression analysis based on 188 TROAP-derived genes | | | | |
| --- | --- | --- | --- | --- |
| id | HR | HR.95L | HR.95H | pvalue |
| WNT5B | 1.160886 | 1.051961 | 1.281091 | 0.003001 |
| WDHD1 | 1.409149 | 1.134824 | 1.749786 | 0.001903 |
| VWA5A | 0.779849 | 0.655198 | 0.928214 | 0.005138 |
| VRK1 | 1.480097 | 1.110594 | 1.972537 | 0.007456 |
| UHRF1 | 1.275724 | 1.085112 | 1.499819 | 0.003185 |
| TUBB | 1.986427 | 1.451675 | 2.718166 | 1.79E-05 |
| TTK | 1.261768 | 1.073578 | 1.482947 | 0.00478 |
| TRIP13 | 1.33722 | 1.106298 | 1.616342 | 0.002661 |
| TPX2 | 1.251103 | 1.06726 | 1.466615 | 0.005732 |
| TPSB2 | 0.836608 | 0.764385 | 0.915655 | 0.000108 |
| TPSAB1 | 0.833486 | 0.76243 | 0.911165 | 6.17E-05 |
| TONSL | 1.682927 | 1.323075 | 2.140651 | 2.23E-05 |
| TMEM97 | 1.331216 | 1.09957 | 1.611663 | 0.003356 |
| TMEM201 | 1.871061 | 1.399236 | 2.501987 | 2.38E-05 |
| TMEM176B | 0.82411 | 0.749273 | 0.90642 | 6.81E-05 |
| TMEM176A | 0.817529 | 0.738765 | 0.90469 | 9.71E-05 |
| TICRR | 1.323796 | 1.081051 | 1.621047 | 0.006647 |
| TFDP1 | 1.343571 | 1.082845 | 1.667073 | 0.007295 |
| TBC1D31 | 1.514231 | 1.126017 | 2.036287 | 0.006046 |
| STMN1 | 1.411635 | 1.156679 | 1.722789 | 0.000694 |
| SPDL1 | 1.368843 | 1.083689 | 1.72903 | 0.008431 |
| SPAG5 | 1.266652 | 1.059015 | 1.514999 | 0.009663 |
| SMOC2 | 0.852243 | 0.778215 | 0.933313 | 0.000564 |
| SLC7A5 | 1.255404 | 1.084867 | 1.452749 | 0.002262 |
| SERPING1 | 0.812552 | 0.730153 | 0.904248 | 0.000142 |
| SERINC2 | 1.163399 | 1.051321 | 1.287425 | 0.003408 |
| SELP | 0.792383 | 0.673252 | 0.932593 | 0.005118 |
| SELENOM | 0.761427 | 0.645697 | 0.897901 | 0.001194 |
| SCX | 1.12107 | 1.041517 | 1.2067 | 0.002341 |
| SCRG1 | 0.863 | 0.776637 | 0.958967 | 0.006167 |
| SAPCD2 | 1.260314 | 1.100848 | 1.44288 | 0.000802 |
| RFC5 | 1.514523 | 1.147479 | 1.998975 | 0.003374 |
| RECQL4 | 1.325236 | 1.094126 | 1.605162 | 0.003977 |
| RCC2 | 1.542751 | 1.174402 | 2.026633 | 0.00184 |
| RCC1 | 1.777438 | 1.38388 | 2.282919 | 6.66E-06 |
| RBL1 | 1.477279 | 1.126785 | 1.936797 | 0.004746 |
| RANBP1 | 1.681156 | 1.261999 | 2.23953 | 0.000385 |
| RAI2 | 0.845571 | 0.750481 | 0.952708 | 0.005853 |
| RAD54L | 1.320376 | 1.116557 | 1.561401 | 0.001159 |
| RAD51AP1 | 1.315218 | 1.078338 | 1.604133 | 0.006844 |
| RAD18 | 1.545115 | 1.165844 | 2.047771 | 0.002464 |
| RAC3 | 1.25878 | 1.121454 | 1.412921 | 9.43E-05 |
| PYCR1 | 1.238989 | 1.106021 | 1.387944 | 0.000216 |
| PXMP2 | 1.476503 | 1.199393 | 1.817638 | 0.000238 |
| PSAT1 | 1.304432 | 1.142453 | 1.489377 | 8.54E-05 |
| PRELP | 0.898463 | 0.833254 | 0.968775 | 0.00535 |
| POLE | 1.456916 | 1.139913 | 1.862076 | 0.002647 |
| POLD2 | 1.900798 | 1.408689 | 2.56482 | 2.65E-05 |
| POLD1 | 1.656971 | 1.245099 | 2.205088 | 0.000533 |
| PLOD2 | 1.267664 | 1.084946 | 1.481152 | 0.002821 |
| PLK1 | 1.252646 | 1.062188 | 1.477256 | 0.00743 |
| PLCXD1 | 1.421719 | 1.170623 | 1.726674 | 0.000387 |
| PIK3IP1 | 0.704709 | 0.592669 | 0.83793 | 7.45E-05 |
| PFAS | 1.340879 | 1.098609 | 1.636576 | 0.003915 |
| PAFAH1B3 | 1.307586 | 1.105562 | 1.546525 | 0.001737 |
| P2RY13 | 0.787089 | 0.669441 | 0.925411 | 0.003751 |
| ORC1 | 1.417316 | 1.17106 | 1.715355 | 0.000342 |
| NT5DC2 | 1.473461 | 1.241886 | 1.748218 | 8.86E-06 |
| NKX6-1 | 1.182542 | 1.065931 | 1.31191 | 0.001549 |
| NDC1 | 1.946654 | 1.4539 | 2.60641 | 7.71E-06 |
| NCAPH | 1.292491 | 1.094019 | 1.526968 | 0.002558 |
| NASP | 1.559845 | 1.193701 | 2.038296 | 0.001125 |
| NAPSB | 0.830341 | 0.728367 | 0.94659 | 0.005419 |
| NAALADL1 | 0.754725 | 0.628272 | 0.906628 | 0.002633 |
| MYLK2 | 1.238671 | 1.057899 | 1.450334 | 0.007831 |
| MYL9 | 0.864647 | 0.787189 | 0.949728 | 0.002389 |
| MYBL2 | 1.295247 | 1.11877 | 1.499562 | 0.000537 |
| MSH2 | 1.541323 | 1.207158 | 1.967991 | 0.000521 |
| MFAP2 | 1.203014 | 1.069027 | 1.353795 | 0.002156 |
| MCM4 | 1.408298 | 1.144103 | 1.733502 | 0.001238 |
| MCM3 | 1.421461 | 1.119413 | 1.805008 | 0.003908 |
| MCM2 | 1.266475 | 1.059555 | 1.513804 | 0.009444 |
| MCM10 | 1.387153 | 1.13641 | 1.693222 | 0.001295 |
| MAD2L2 | 1.466227 | 1.166519 | 1.842937 | 0.001038 |
| MAD2L1 | 1.36644 | 1.107335 | 1.686173 | 0.00361 |
| LTB | 0.812353 | 0.71445 | 0.923671 | 0.001515 |
| LRRC17 | 1.142162 | 1.046174 | 1.246958 | 0.002999 |
| LOXL2 | 1.195539 | 1.046015 | 1.366436 | 0.008795 |
| LMOD1 | 0.918638 | 0.863935 | 0.976805 | 0.006746 |
| LMNB2 | 1.482228 | 1.201923 | 1.827903 | 0.000234 |
| LIMS2 | 0.761855 | 0.676571 | 0.857889 | 7.11E-06 |
| LIG1 | 1.431198 | 1.126136 | 1.8189 | 0.003376 |
| KLRB1 | 0.777385 | 0.655621 | 0.921764 | 0.003764 |
| KIFC1 | 1.238601 | 1.060232 | 1.446978 | 0.006993 |
| KIF4A | 1.227909 | 1.056399 | 1.427263 | 0.007479 |
| KIF2C | 1.240321 | 1.068789 | 1.439383 | 0.004569 |
| KIF15 | 1.301332 | 1.091752 | 1.551145 | 0.003285 |
| KCND3 | 0.807564 | 0.718786 | 0.907307 | 0.000322 |
| JPT1 | 1.468127 | 1.185279 | 1.818472 | 0.000437 |
| ITPRIPL1 | 1.430752 | 1.180849 | 1.733542 | 0.000255 |
| ITGB3BP | 1.737953 | 1.254593 | 2.407537 | 0.000887 |
| ITGA10 | 1.191954 | 1.086885 | 1.30718 | 0.000192 |
| IRAG1 | 0.870904 | 0.796413 | 0.952363 | 0.002447 |
| ILF2 | 1.919449 | 1.392364 | 2.646063 | 6.87E-05 |
| IL33 | 0.871462 | 0.796577 | 0.953387 | 0.002689 |
| IGLC3 | 0.913987 | 0.854269 | 0.977879 | 0.009086 |
| IGLC2 | 0.911321 | 0.851531 | 0.97531 | 0.007318 |
| IGKC | 0.917302 | 0.861132 | 0.977137 | 0.007421 |
| IGHM | 0.905244 | 0.843826 | 0.971133 | 0.005485 |
| IGHG2 | 0.907642 | 0.847083 | 0.972531 | 0.00595 |
| IGHG1 | 0.919728 | 0.864089 | 0.978949 | 0.008584 |
| IGF2BP3 | 1.262673 | 1.093828 | 1.457582 | 0.00145 |
| IGF2BP1 | 1.231994 | 1.073854 | 1.413422 | 0.002915 |
| HPGD | 0.783365 | 0.679978 | 0.902471 | 0.000722 |
| HMGN2P5 | 1.316974 | 1.078056 | 1.608842 | 0.007021 |
| HMGN2P3 | 1.360803 | 1.12598 | 1.644598 | 0.001434 |
| HMGN2 | 1.404223 | 1.112831 | 1.771916 | 0.004225 |
| HMGB3 | 1.518111 | 1.269024 | 1.816089 | 4.98E-06 |
| HAS2 | 1.181967 | 1.057166 | 1.3215 | 0.003321 |
| GPR3 | 1.359808 | 1.115223 | 1.658034 | 0.002382 |
| GPC2 | 1.521272 | 1.264607 | 1.830031 | 8.59E-06 |
| GMNN | 1.3805 | 1.097988 | 1.735702 | 0.005777 |
| GINS4 | 1.385406 | 1.123085 | 1.708996 | 0.002336 |
| GINS3 | 1.482122 | 1.110289 | 1.978481 | 0.007589 |
| GINS2 | 1.35519 | 1.103806 | 1.663824 | 0.003691 |
| GINS1 | 1.292656 | 1.066425 | 1.566879 | 0.008919 |
| GABRA3 | 1.20539 | 1.064429 | 1.365019 | 0.00324 |
| G2E3 | 1.467748 | 1.117724 | 1.927384 | 0.005769 |
| FMOD | 0.895993 | 0.831366 | 0.965644 | 0.004037 |
| FHAD1 | 1.303262 | 1.094417 | 1.55196 | 0.002954 |
| FGF7 | 0.86441 | 0.791029 | 0.944599 | 0.001286 |
| FCER1A | 0.743962 | 0.645444 | 0.857516 | 4.49E-05 |
| FBN2 | 1.19168 | 1.077565 | 1.317881 | 0.000639 |
| FAM72D | 1.549612 | 1.142521 | 2.101754 | 0.00485 |
| FAM72B | 1.399663 | 1.092047 | 1.793931 | 0.007922 |
| EVA1C | 0.78622 | 0.680926 | 0.907796 | 0.001043 |
| ETV4 | 1.195722 | 1.07512 | 1.329852 | 0.000983 |
| ESPL1 | 1.323969 | 1.091614 | 1.605781 | 0.004368 |
| ERCC6L | 1.276911 | 1.060846 | 1.536984 | 0.009753 |
| ENO1 | 1.375281 | 1.138553 | 1.66123 | 0.000945 |
| DRAXIN | 1.219893 | 1.087482 | 1.368426 | 0.000697 |
| DNMT3B | 1.482705 | 1.207031 | 1.82134 | 0.000175 |
| DLX1 | 1.216851 | 1.095855 | 1.351206 | 0.00024 |
| DLGAP5 | 1.251188 | 1.078686 | 1.451276 | 0.00307 |
| CTSG | 0.809541 | 0.73155 | 0.895846 | 4.35E-05 |
| CTHRC1 | 1.153355 | 1.043841 | 1.274358 | 0.005065 |
| CPXM2 | 0.86915 | 0.792988 | 0.952628 | 0.002725 |
| CPA3 | 0.827037 | 0.754597 | 0.906431 | 4.90E-05 |
| COL24A1 | 1.208789 | 1.046561 | 1.396165 | 0.009911 |
| CLU | 0.861585 | 0.789715 | 0.939996 | 0.000801 |
| CLEC10A | 0.7857 | 0.696695 | 0.886077 | 8.43E-05 |
| CIP2A | 1.379689 | 1.126141 | 1.690324 | 0.001893 |
| CHTF18 | 1.388588 | 1.106353 | 1.742823 | 0.004629 |
| CHEK1 | 1.425938 | 1.123152 | 1.810352 | 0.003573 |
| CEP72 | 1.349295 | 1.076228 | 1.691644 | 0.009412 |
| CENPO | 1.42465 | 1.124941 | 1.804208 | 0.003315 |
| CENPI | 1.372406 | 1.12763 | 1.670315 | 0.001586 |
| CENPA | 1.250973 | 1.056102 | 1.481801 | 0.009549 |
| CDK1 | 1.249479 | 1.060833 | 1.471672 | 0.00765 |
| CDCA8 | 1.232261 | 1.060578 | 1.431735 | 0.006367 |
| CDCA4 | 1.394614 | 1.133344 | 1.716116 | 0.001675 |
| CDC25A | 1.502596 | 1.217201 | 1.854906 | 0.000151 |
| CDC20 | 1.189728 | 1.04504 | 1.354449 | 0.008643 |
| CCNF | 1.390673 | 1.137307 | 1.700483 | 0.00131 |
| CCNA2 | 1.261272 | 1.068277 | 1.489134 | 0.006155 |
| CCL19 | 0.875677 | 0.793223 | 0.966702 | 0.00851 |
| CCL17 | 0.795409 | 0.675276 | 0.936915 | 0.006144 |
| CCDC69 | 0.729859 | 0.642883 | 0.828602 | 1.15E-06 |
| CCDC18 | 1.430425 | 1.096652 | 1.865783 | 0.008278 |
| CBX7 | 0.760482 | 0.647832 | 0.892722 | 0.000816 |
| CAVIN4 | 1.246695 | 1.090629 | 1.425094 | 0.001232 |
| CA9 | 1.116946 | 1.030264 | 1.210921 | 0.007289 |
| C1S | 0.794148 | 0.712408 | 0.885268 | 3.20E-05 |
| C1R | 0.803993 | 0.706035 | 0.915543 | 0.000998 |
| C19orf48 | 1.572332 | 1.228214 | 2.012863 | 0.000329 |
| C18orf54 | 1.421858 | 1.116259 | 1.811123 | 0.004361 |
| C11orf96 | 0.86665 | 0.783303 | 0.958865 | 0.005535 |
| BLM | 1.437 | 1.123304 | 1.838299 | 0.00391 |
| BHMT2 | 0.862819 | 0.775259 | 0.960268 | 0.006881 |
| BCL2L12 | 1.536411 | 1.15891 | 2.036877 | 0.002835 |
| AURKB | 1.284795 | 1.114139 | 1.481591 | 0.000568 |
| AUNIP | 1.467256 | 1.181221 | 1.822556 | 0.00053 |
| ATAD3A | 1.628325 | 1.264823 | 2.096294 | 0.000155 |
| ASPA | 0.82401 | 0.718972 | 0.944394 | 0.005398 |
| ARSJ | 1.268649 | 1.066241 | 1.509481 | 0.007292 |
| AOC3 | 0.860659 | 0.78751 | 0.940602 | 0.000929 |
| ANP32E | 1.374274 | 1.080563 | 1.747819 | 0.009554 |
| ALDH1A1 | 0.772463 | 0.694165 | 0.859593 | 2.20E-06 |
| AL590617.2 | 1.303327 | 1.147464 | 1.480362 | 4.57E-05 |
| AL442125.2 | 1.392406 | 1.141399 | 1.698613 | 0.001099 |
| ADGRD1 | 0.863489 | 0.778891 | 0.957275 | 0.005271 |
| ACTL6A | 1.612733 | 1.200893 | 2.165812 | 0.001489 |
| ACKR1 | 0.855581 | 0.778793 | 0.93994 | 0.00115 |
| AC112777.1 | 1.327255 | 1.09556 | 1.607949 | 0.003824 |
| AC069499.1 | 1.353549 | 1.118108 | 1.638566 | 0.001903 |
| AC027031.2 | 1.422476 | 1.200505 | 1.68549 | 4.68E-05 |
| AC012073.1 | 1.429895 | 1.122989 | 1.820676 | 0.003721 |
| AC011503.2 | 1.409581 | 1.092629 | 1.818476 | 0.008251 |

**Table S2.** The primer sequences used for RT-qPCR and siRNA.

| Gene | Primer sequence (5′-3′) |
| --- | --- |
| TROAP-NC (sense) | UUCUCCGAACGUGUCACGUTT |
| TROAP-NC (antisense) | ACGUGACACGUUCGGAGAATT |
| TROAP-siRNA (sense) | GGAGAGUUGUAUAAGGUCATT |
| TROAP-siRNA(antisense) | UGACCUUAUACAACUCUCCTT |
| TROAP(F) | GTCAGGAGAAAAGCGGAGGAAGC |
| TROAP(R) | CGTGCGTTTCTGAGAGCGTACC |
| GAPDH(F) | CAAGGTCATCCATGACAACTTTG |
| GAPDH(R) | GTCCACCACCCTGTTGCTGTAG |
